# Supplementary material for: A Novel Ethanolic Two-Phase System Based on Deep Eutectic Solvents and Amphiphilic Copolymer for the Extraction of Neohesperidin and Naringin from the Pomelo Peel
Source: Foods. 2022 Aug 26;11(17):2590. doi: 10.3390/foods11172590 (PMC9455264; doi:10.3390/foods11172590)
Supplement: Supplementary file 1 [file foods-11-02590-s001.zip › foods-1880746-supplementary.pdf]

Supplementary Data

A novel ethanolic two-phase system based on deep eutectic solvents and amphiphilic copolymer for the extraction of neohesperidin and naringin from the pomelo peel

Shanshan Wang [1], Zongkui Qin [1], Yicong Wang [1], Leilei Liu [1,*], Zhijian Tan [2,*]

[1] *Key Laboratory of Hunan Forest Products and Chemical Industry Engineering, College of Chemistry and Chemical Engineering, Jishou University, Zhangjiajie 427000, China*

[2] *Institute of Bast Fiber Crops and Center of Southern Economic Crops, Chinese Academy of Agricultural Sciences, Changsha 410205, China*

*Corresponding authors:

E-mail addresses: leileiliu2019@jsu.edu.cn (L. Liu); tanzhijian@caas.cn (Z. Tan).

Table Captions

Table S1 The chemical structures of HBAs and HBDs.

Table S2 Experimental (liquid+liquid) equilibrium mass fractions (binodal curve data) for the system N_{4444}Br-Gly (X)+PL 64 (Y) at 25 °C and atmospheric pressure.

Table S3 The volumes of PL 64-rich and DESs-rich phases for different HBAs to HBDs molar ratio of DESs (3.5g, 29wt% N_{4444}Br-Gly/61wt%PL 64).

Table S4 Preparation method of phosphoric acid buffer solutions.

Table S5 Preparation method of ethanol with diverse pH.

Figure Captions

Figure S1 *K* values for Nar and Neo in different ETPSs. The extraction conditions were as follows: (a) DESs of 30 wt%, PL 64 of 35 wt%, temperature of 25 °C, (b) DESs of 43 wt%, PL 64 of 43 wt%, temperature of 25 °C.

Figure S2 Effects of the (a) the mass ratio of DESs and PL 64, (b) DES concentration, (c) PL 64 concentration, (d) molar ratio of HBAs and HBDs, and (e) equilibrium temperature on the *K* values. The extraction conditions were as follows: (a) temperature of 25 °C, (b) PL 64 of 61 wt%, temperature of 25 °C, (c) DESs of 29 wt%, temperature of 25 °C, (d) DESs of 29 wt%, PL 64 of 61 wt%, temperature of 25 °C, (e) DESs of 29 wt%, PL 64 of 61 wt%.

Figure S3 Effects of the (a) buffer solutions with diverse pH and (b) ethanol with diverse pH on the *K* values. The extraction conditions were as follows: DESs of 29 wt%, PL 64 of 61 wt%, temperature of 25 °C.

Figure S4 The chromatograms of (a) standard DESs, (b) recovered DESs. (c) Standard fitting curves for DESs. The HPLC used is the same as that used to detect NAR and Neo. The mobile phase and detection wavelength were 40:60 of acetonitrile/0.1% phosphoric acid and 210 nm, respectively.

Figure S5 Chromatograms of (a) standard compounds, (b) PL 64-rich phase, and (c) DESs-rich phase, (1-Na, 2-Neo). (d) Standard curves of Nar and Neo, the linear ranges for Nar and Neo were 0.80-63.92 and 0.49-63.80 μg/mL, respectively.

Table S1 The chemical structures of HBAs and HBDs

HBAs	Chemical structure	HBDs	Chemical structure
Choline chloride	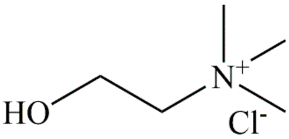	D- (+)-Maltose monohydrate	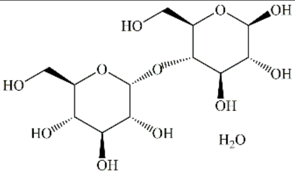
Betaine	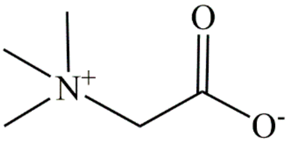	D- (+)-Glucose	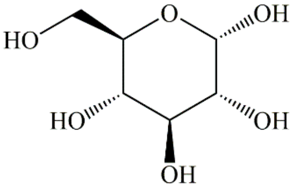
L- (-)-Proline	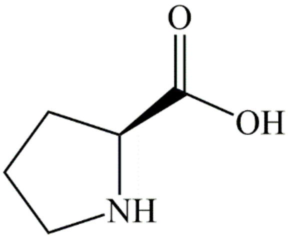	Oxalic acid	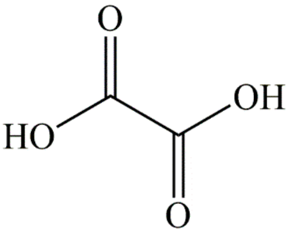
Tetraethylammonium chloride	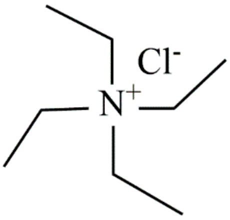	Glycerol	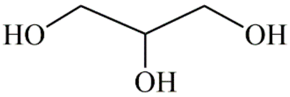
Tetrabutylammonium chloride	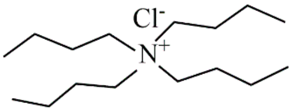	Xylitol	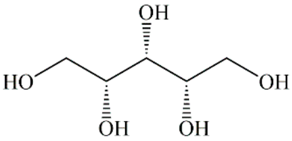
Tetraethlammonium bromide	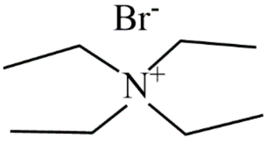		
Tetrapropylammonium bromide	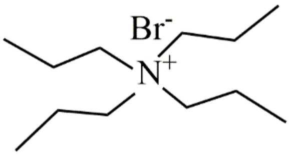		
Tetrabutylammonium bromide	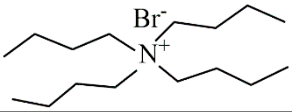		

Table S2 Experimental (liquid+liquid) equilibrium mass fractions (binodal curve data) for the system N_{4444}Br-Gly (X)+PL 64 (Y) at 25 °C and atmospheric pressure.

**System Composition**		**Final Composition**	
X_1 (%)	Y_1 (%)	X_2 (%)	Y_2 (%)
94.82	5.18	74.07	4.05
89.89	10.11	69.14	7.78
85.00	15.00	65.49	11.56
78.24	21.76	61.73	17.17
75.04	24.96	60.68	20.19
69.39	30.61	56.10	24.74
64.80	35.20	51.66	28.06
60.11	39.89	48.92	32.47
54.30	45.70	44.56	37.50
50.17	49.83	41.66	41.37
44.92	55.08	37.12	45.52
40.26	59.74	33.69	49.99
35.39	64.61	29.95	54.68
29.70	70.30	25.46	60.27
25.48	74.52	22.61	66.13
20.17	79.83	18.08	71.55
15.62	84.38	14.26	77.04
10.20	89.80	9.58	84.32
4.99	95.01	4.83	91.92
20.30	79.70	17.96	70.51
15.03	84.97	13.51	76.34
11.12	88.88	10.26	81.98
6.06	93.94	5.64	87.53

Table S3 The volumes of PL 64-rich and DESs-rich phases for different HBAs to HBDs molar ratio of DESs (3.5g, 29wt% N_{4444}Br-Gly/61wt%PL 64).

**HBAs:HBDs**	$V_{PL\ 64\text{-rich}}$	$V_{DESs\text{-rich}}$
1:3	2.60	0.80
1:4	2.60	0.75
1:5	2.60	0.80
1:6	2.65	0.75
1:7	2.65	0.75

Table S4 Preparation method of phosphoric acid buffer solutions.

pH	0.2M$K_2HPO_4 \cdot 3H_2O$ (mL)	0.1M $C_6H_8O_7$ (mL)
3	4.11	15.89
4	7.71	12.29
5	10.30	9.7
6	12.63	7.37
7	16.47	3.53
8	19.45	0.55

Table S5 Preparation method of ethanol with diverse pH.

pH	$C_6H_8O_7$ or NaOH (g)	Ethanol (mL)
1.24	10.00 ($C_6H_8O_7$)	30
3.16	0.01 ($C_6H_8O_7$)	15
5.34	0.001 ($C_6H_8O_7$)	130
13.78	0.015 (NaOH)	25

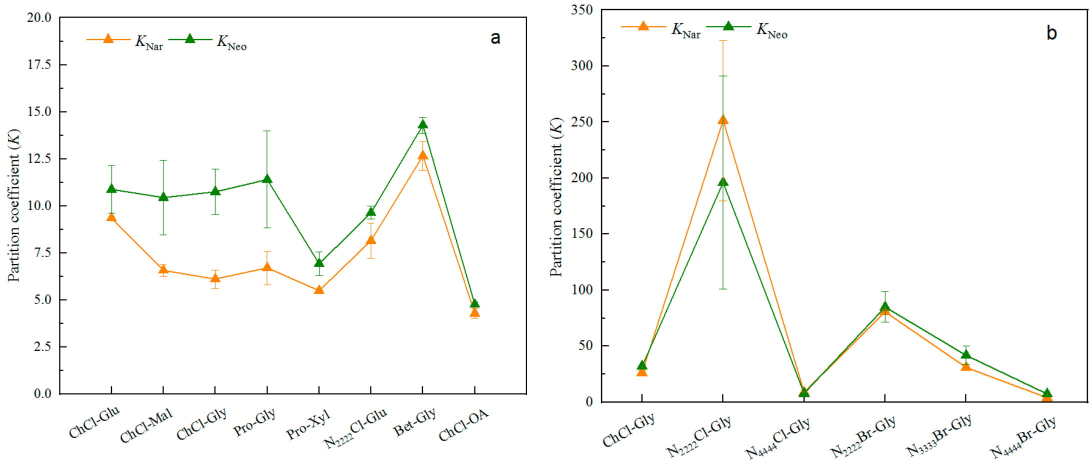

Figure S1 K values for Nar and Neo in different ETPSs. The extraction conditions were as follows: (**a**) DESs of 30 wt%, PL 64 of 35 wt%, temperature of 25 °C, (**b**) DESs of 43 wt%, PL 64 of 43 wt%, temperature of 25 °C.

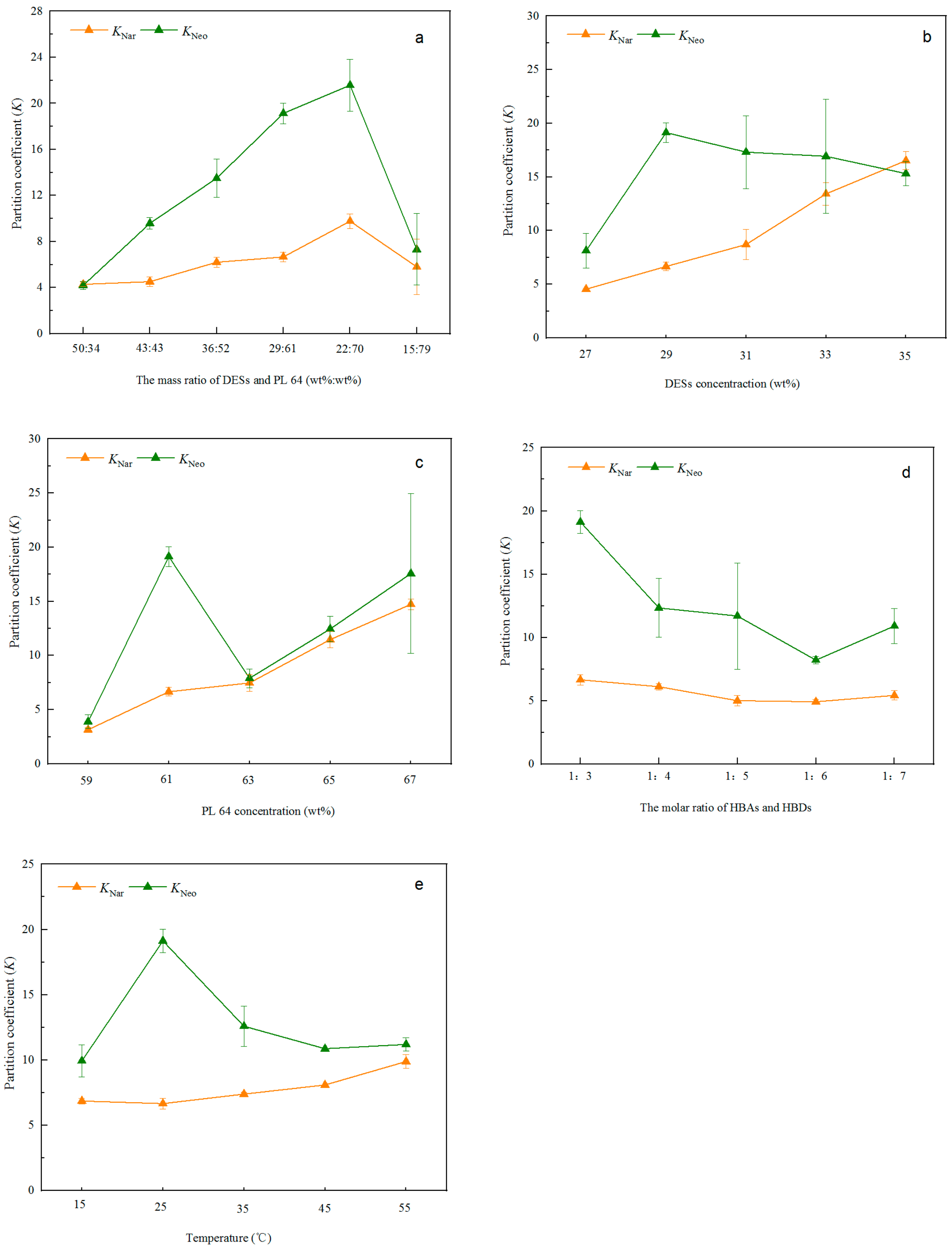

Figure S2 Effects of the (a) the mass ratio of DESs and PL 64, (b) DES concentration, (c) PL 64 concentration, (d) molar ratio of HBAs and HBDs, and (e) equilibrium temperature on the K values. The extraction conditions were as follows: (a) temperature of 25 °C, (b) PL 64 of 61 wt%, temperature of 25 °C, (c) DESs of 29 wt%, temperature of 25 °C, (d) DESs of 29 wt%, PL 64 of 61 wt%, temperature of 25 °C, (e) DESs of 29 wt%, PL 64 of 61 wt%.

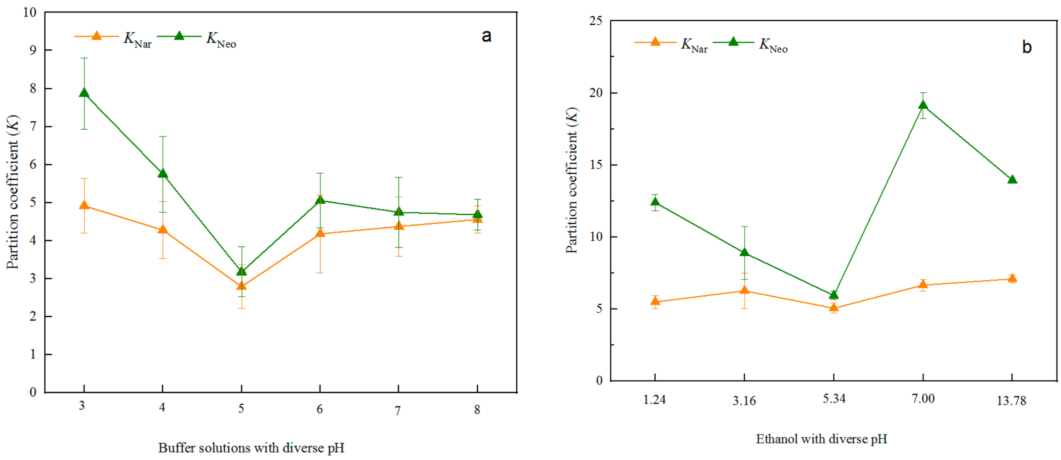

Figure S3 Effects of the (a) buffer solutions with diverse pH and (b) ethanol with diverse pH on the K values. The extraction conditions were as follows: DESs of 29 wt%, PL 64 of 61 wt%, temperature of 25 °C.

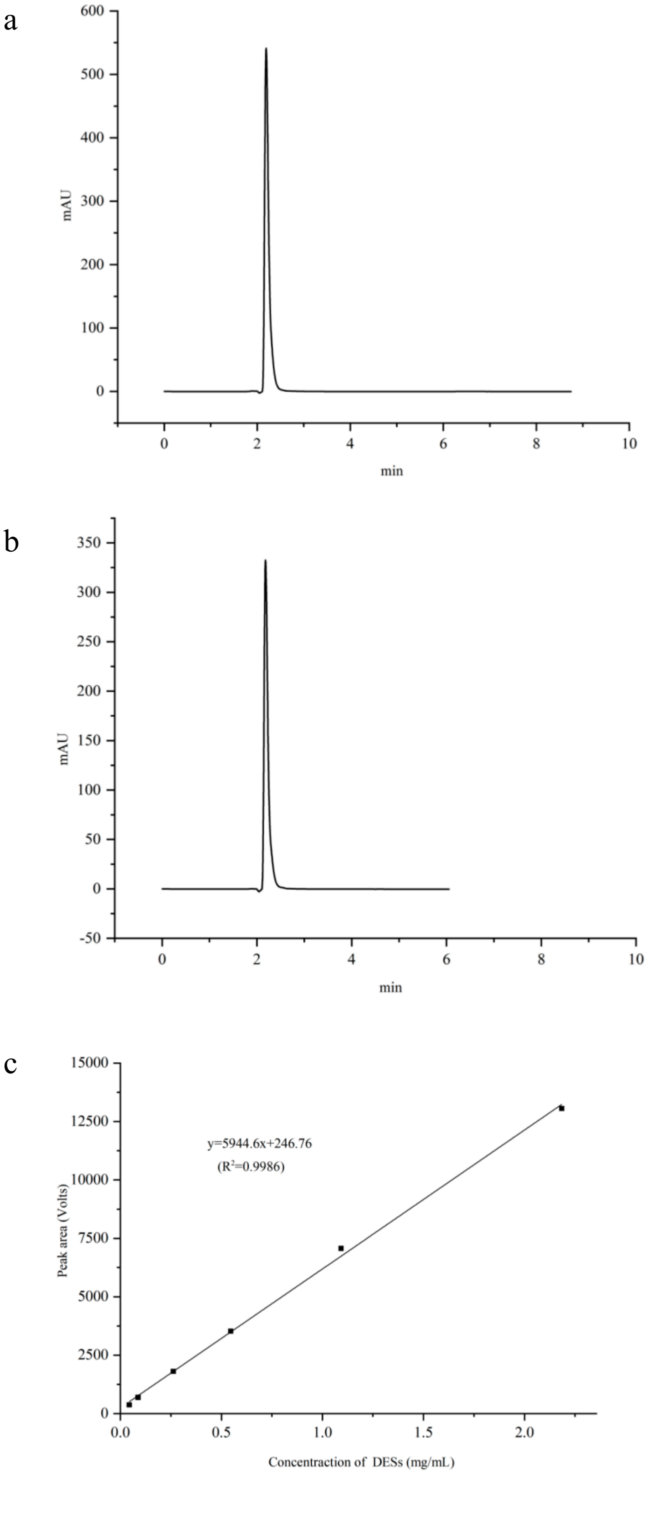

Figure S4 The chromatograms of (a) standard DESs, (b) recovered DESs. (c) Standard fitting curves for DESs. The HPLC used is the same as that used to detect NAR and Neo. The mobile phase and detection wavelength were 40:60 of acetonitrile/0.1% phosphoric acid and 210 nm, respectively.

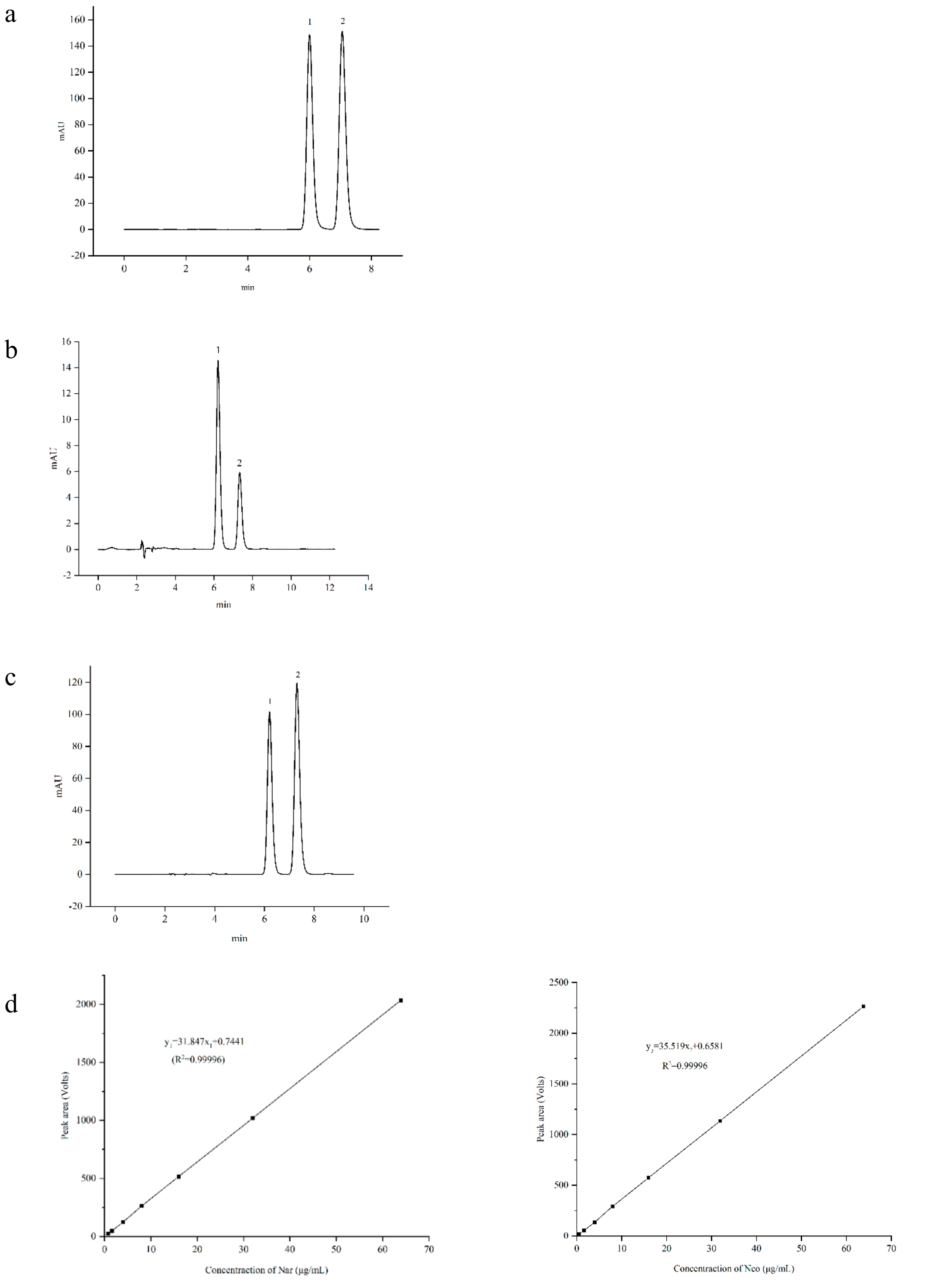

Figure S5 Chromatograms of (a) standard compounds, (b) PL 64-rich phase, and (c) DESs-rich phase, (1-Na, 2-Neo). (d) Standard curves of Nar and Neo, the linear ranges for Nar and Neo were 0.80-63.92 and 0.49-63.80 μg/mL, respectively.
